# Increased activity in broiler chickens is associated with better feed conversion

**DOI:** 10.1016/j.psj.2026.106599

**Published:** 2026-02-07

**Authors:** Christl A. Donnelly, Stephen A. Ellwood, Stephen J. Roberts, Marian Stamp Dawkins

**Affiliations:** a Department of Statistics, University of Oxford, UK; b Department of Biology, University of Oxford, UK; c Department of Engineering Science, University of Oxford, UK

**Keywords:** Broiler, Activity, Welfare, Feed conversion, Mortality

## Abstract

Farmers are understandably concerned that many proposed improvements to broiler chicken welfare such as ‘enrichments’ lead to the birds being more active, eating more and therefore result in financially detrimental effects on Feed Conversion Ratio (FCR). The current evidence is inconclusive, but most research so far has relied on small-scale pen studies, not flocks studied under commercial conditions. We measured the life-long activity of 34 commercial flocks of Cobb broilers using smart camera technology and analyzed the data using four statistical descriptors of the patterns made by flock movements – mean, variance, skew and kurtosis of optical flow. For each day, we scored each flock by its scaled deviation from the median for each of the four descriptors and gave it 4 overall activity scores, based on its average lifetime deviation from median ([average optical flow value – median]/√median). The results showed that, contrary to widespread concerns, FCR was no higher in more active flocks and that on average more active flocks tended to have lower (i.e. more efficient) FCR (*p* = 0.060). There were positive correlations between FCR and the lifetime activity score using both the skew of optical flow (*r* = 0.608, *p* < 0.001) and kurtosis (*r* = 0.603, *p* < 0.001), both suggesting that increasing numbers of active birds within a flock were associated with lower FCR. There were also positive correlations between skew and kurtosis of optical flow and mortality (*r* = 0.388, *p* = 0.023 and *r* = 0.454; *p* = 0.007) respectively), as well as an even higher correlation between FCR and mortality (*r* = 0.698; *p* < 0.001), which suggests that the favorable effect of activity on FCR may at least in part, be via decreased mortality. While not all welfare improvement may result in improvements in FCR, these results show that increased flock activity is not itself the problem that might be feared.

## Introduction

Farmers around the world are increasingly having to prioritize productivity and efficiency in order to stay in business (McKinsey, 2024), which means that they may face a conflict between what is financially viable and what they see as the costs of improving animal welfare. For example, ‘enrichments’ such as higher light levels, perches, platforms, objects to peck at, straw bales, shelters etc. that are primarily designed to improve chicken welfare may raise concerns that they will have detrimental effects on efficiency (Jones et al., 2020). Not only do many enrichments cost money to buy, maintain and clean, they also take up floor space and part of their welfare advantage is openly stated to be that they encourage birds to become more active and perform more of their natural behaviour patterns (Bizeray et al., 2000; Bach et al., 2019; Vasdal et al., 2019). Since activity stimulates birds to eat more (Bizeray et al., 2002; Rayner et al., 2020) and feed is the largest single cost in broiler production (Aviagen, 2025), farmers are understandably wary of anything – including lighting programs and enrichments – that increases how much birds eat.

The belief that welfare improvements make birds more active but also adversely affect the commercially sensitive production metric of Feed Conversion Ratio (FCR= feed consumed by a flock/ weight gained) is therefore potentially a serious obstacle to the widespread uptake of welfare measures, such as enrichments, for broiler chickens. However, it does not follow that if broilers move more and use more energy this will necessarily result in a higher (i.e. less efficient) FCR because FCR, as used by poultry farmers, is a flock-level measure that depends critically not just on how much feed individual birds consume but also on their mortality. A flock consisting of individuals that were all highly efficient converters of feed into meat could show a very inefficient FCR if half of them died in the last week, because so much food would be wasted. On the other hand, a flock consuming a relatively large amount of feed through being very active could, in the end, show a lower and more efficient FCR if most of the birds had better health and lower mortality.

The current evidence on how activity affects FCR is difficult to interpret because studies differ substantially in scale and content (pen trials v. commercial flocks, enrichment type, genetics and outcome measures (Meyer et al., 2019; Dawson et al., 2021; Zhou et al., 2024; Kyriazakis et al., 2025)). An increasing number of studies are showing that some enrichments result in more movement and improved welfare while not having any detrimental effect on productivity and in some cases may even result in a lower (i.e. more efficient) FCR (Meyer et al., 2019; Vasdal et al., 2019; Yang et al., 2020; Zahoor et al., 2022; Shynkaruk et al., 2024; Ghani et al., 2025; Zago-Dias et al., 2025). However, most of the studies reported so far have been carried out using small-scale pen trials, not commercial broiler flocks, where the real effects on FCR may be quite different.

We here report the results of recording activity from commercial broiler flocks and relating this to FCR and mortality in a way that is directly relevant to commercial farming. We used smart technology cameras to measure activity levels so that we could record data over the entire lifetime of the flock and we used multiple measures of flock activity to conclude that on average more active flocks had lower FCR.

## Materials and methods

### Birds and Housing

As part of a larger study described elsewhere (Dawkins et al., 2025), data were collected from a commercial broiler farm in south-eastern USA. 34 commercial broiler flocks over 12 cycles were studied between December 2020 and November 2023. The farm had 4 identical modern houses (12.8 m x 122 m), each equipped with standard feeders, waterlines and brooders (12.8 m x 122 m) with wood shavings as litter. Two houses had a 60 cm wide strip of clear plastic running the length of the houses 180 cm high on the sidewalls. This allowed for natural light to enter the houses but could be sealed to convert the house to artificial light only.

For each flock cycle, between 16,800 and 19,200 mixed sex one-day-old Cobb broilers (average=18,800) were placed in each house and grown to 52-54 days without thinning (average weight = 3.61 kg). During the course of the study, the farm management made a number of changes, including varying the lighting (5 lux, 20 lux, natural and gradient, with lighting equipment from different manufacturers) and the number of enrichments (triangular huts at 0, 1/1000, 3/1000 and 6/1000 birds), in various combinations, over which we had no control.

### ***On-farm measurements*** (supplied by the producer company)

Farm staff recorded daily and total mortality. Estimated feed consumption was routinely recorded from house-specific meters, converted to lbs/1000 birds and used to calculate total feed consumed by each flock.

### ***Processing plant measurements*** (supplied by the producer company)

Body weights at clearance received by the respective processing plants were recorded by the staff there and, together with the total amount of food consumed during the life of a flock, were used to calculate Feed Conversion Ratios (FCR) as

FCR (unadjusted) = total feed consumed/ (number of birds sold x average weight).

### Recording flock activity

In each house, 2 Hikvision cctv IP (Internet Protocol) zoom cameras (DS-2CD2646G2-IZS) were ceiling-mounted facing vertically down and their horizontal field of view (FOV) adjusted to 3 m. As far as possible, the cameras were positioned so that their FOVs captured bird behaviour between feeder or drinker lines. Cameras streamed footage continuously throughout the life of a flock to a single-board computer (SBC; Raspberry Pi) situated in the ante-room of the broiler house via a wired connection. Technical problems with the equipment meant that some optical flow records were incomplete or the number of frames dropped below the expected 3600 frames/15-minute period at which the cameras were set to record. All data were therefore filtered to eliminate any sequences with unreliable frame numbers. Flocks with no records in the first week were also eliminated from the analysis. This resulted in useable data from only *n* = 34 flocks.

### Image processing

Image processing took place locally on the SBC connected to each camera. Each video frame was divided into 1200 (40 × 30) 8-by-8 pixel blocks and optical flow (rate of change of image velocity) estimated for each block every 0.25 seconds. These estimated optical flow velocities (OF) were then combined, on a frame-by-frame basis to give the total ‘flow’ over the entire image expressed as the mean, variance, skew and kurtosis of optical flow (Dawkins et al., 2012; Roberts et al., 2012). Data were then averaged over 15 min (median of 3600 frames) and these 15 min summaries were then averaged further to give daily (12 h: 8:00 to 20:00 h) values of mean, variance, skew and kurtosis of optical flow**.** The data from the two cameras was combined by taking the average.

### Optical flow variables

Mean optical flow is a measure of the average level of activity of a flock and so is a good general indicator of how much a flock is moving. Variance of optical flow indicates the variation around this mean. However, further information about the state of a flock can be gathered from the skew and kurtosis of its movement distribution. The skew shows the extent to which the mode is displaced from the mean – that is, whether the bulk of the observations are above or below average. The kurtosis indicates the proportion of measurements that are to be found in the tails (edges) of a distribution compared to the centre and is particularly useful for identifying outliers (see SPSS Tutorial for fuller explanation). Typically, broiler behaviour recorded by optical flow shows a highly positive (left-hand mode) skew, coupled with a highly positive kurtosis (cf. SPSS Tutorials, Score Test 6). This indicates that most of a flock’s movement is clustered towards the low end of the movement distribution with a small number of outlier individuals moving much more. Direct observations made inside broiler houses show that birds typically spend up to 90% of their time sitting and only about 10% of their time actively walking around (Weeks et al., 2000; Dixon, 2020). Active walkers are thus outliers when compared to the relative inactivity of most of the flock most of the time.

Confirmation of this interpretation comes from comparing optical flow output from cameras with frame-by-frame analysis of the behaviour of birds from the same section of video. Both skew and kurtosis correlate negatively with the number of birds actively walking (walking for at least 10 seconds continuously) (Dawkins et al., 2013). To a rough approximation, therefore, the extent of positive skew of optical flow represents the movement of birds sitting or being relatively immobile while the kurtosis on the upper end of the distribution is strongly influenced by the number of actively walking birds. If there are many individuals actively walking around, a walking bird does not stand out from the rest of the flock and the kurtosis will be relatively low. However, if only a few are walking, their movement will look unusual when compared to the relative inactivity of the rest of the flock and the optical flow distribution will show a more pronounced positive kurtosis. Skew and kurtosis thus give different kinds of about flock activity than mean alone and have also been shown to be clearly correlated with key welfare outcomes such as % mortality, hockburn and foot pad dermatitis (Dawkins et al., 2012, 2017, 2021, 2025).

### Statistical analysis

Four separate measures of lifetime flock activity were calculated using the four optical flow variables – mean OF, variance OF, skew OF and kurtosis OF.

First, the median value for each variable on each day was calculated for all 34 flocks. Second, each flock was compared, day by day, to the day-specific median for each variable and its deviation from that median (positive or negative) divided by the square root of the median was used as its score for that day. This square root division was to correct for possible changes in absolute values over the lifetime of a flock. Third, the lifetime average scaled deviation of a flock’s activity from the median as measured by each of the optical flow variables was calculated as

= average ([daily value of variable – median]/√median)

The distribution of each of these measures was assessed to detect any anomalous values. Finally, a Pearson correlation coefficient was calculated for each of the 4 lifetime averages as predictors and both FCR and total mortality as outcomes. Linear regression was also used to demonstrate a linear relationship. In addition, a Pearson correlation coefficient was calculated for the two outcome variables, FCR and total mortality. Given the numerous combinations of lighting and enrichments that the farm management introduced during the course of the trials, it. was not possible to robustly analyze the effects of either lighting or enrichments on flock activity separately. Instead, both were regarded as likely contributors to what was regarded as residual variation in linear models.

## Results

The 34 flocks showed a wide variation in both mortality and FCR (Table 1). There was also variation in lifetime activity levels as described by the mean, variance skew and kurtosis of the OF.

**Table 1:** Mortality, FCR and lifetime activity for the 34 flocks.

	Average	St.Dev.	Range
% Mortality (52d)	9.56	3.58	5.64 to 17.37
FCR	1.91	0.08	1.75 to 2.07
**Lifetime flock average optical flow ([value-median]/√median)**			
Mean OF	0.002	0.126	−0.269 to 0.265
Variance OF	−0.004	0.123	−0.271 to 0.294
Skew OF	−0.133	0.498	−1.275 to 0.521
Kurtosis OF	−0.237	2.308	−5.141 to 3.719

Table 2 shows the correlations between FCR and measures of average lifetime flock activity based on the four optical flow variables.

**Table 2:** Pearson correlation coefficients between the lifetime average scaled deviation of a flock’s activity from the median based four optical flow variables and FCR.

	Pearson correlation	95% CI	P
Mean OF	−0.326	−0.598 to 0.013	0.060
Variance OF	0.131	−0.217 to 0.449	0.462
Skew OF	0.608	0.340 to 0.785	0.0001
Kurtosis OF	0.603	0.333 to 0.782	0.0002

Table 2. Pearson correlation coefficients between the lifetime average scaled deviation of a flock’s activity from the median based four optical flow variables and FCR. Although the correlation between lifetime average scaled deviation of a flock’s mean OF from the median and FCR does not reach the 0.05 significance level, it is striking that the correlation is negative not positive, which means that flocks with the higher mean levels of activity tended to have the lower (i.e. more efficient) FCRs. They were clearly not less efficient.

**Fig. 1:**
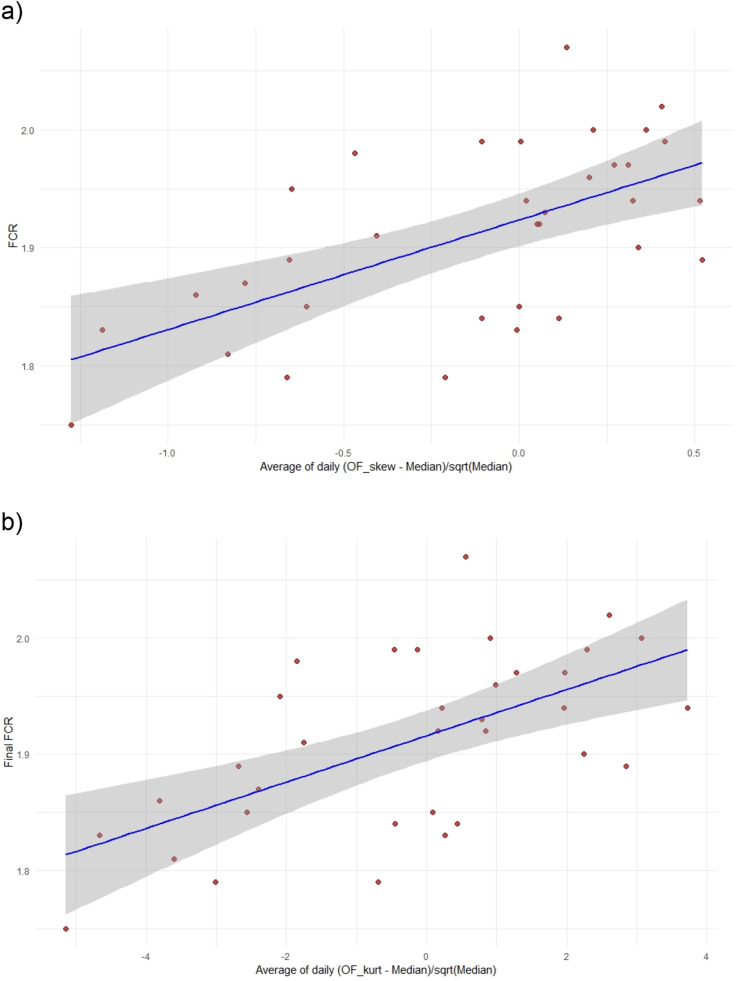
a. Relationship between the OF skew lifetime activity indicator averaged over days 0-50 and FCR. Adjusted R-squared=0.350; *F* = 18.78, df=1,32, *p* = 0.0001. b. Relationship between the OF kurtosis lifetime activity indicator averaged over days 0-50 and FCR. Adjusted R-squared=0.344; *F* = 18.32, df=1,32, *p* = 0.0002.

The explanatory power of the lifetime average scaled deviation of a flock’s activity from the median using the skew- and kurtosis-based score**s** of a flock to predict FCR is further shown by regression analysis (Fig. 1a and b) which show that high-skew and high-kurtosis flocks had higher (less efficient) FCRs. A demonstration of the robustness of these results is given as Supplementary Information.

Table 3 shows the relationships between lifetime average flock activity and mortality. The lifetime activity measure based on mean optical flow shows no evidence that more active flocks have higher mortality. On the contrary, there is a negative (*P* = 0.161) correlation between the lifetime activity score based on mean optical flow and a positive correlation with activity scores using skew and (particularly) kurtosis. Higher skew and kurtosis, both indicative of less active flocks, are thus also associated with higher mortality.

**Table 3:** Pearson correlation coefficients between the lifetime average scaled deviation of a flock’s activity from the median for the four optical flow variables and mortality.

	Pearson correlation	95% CI	P
Mean OF	−0.246	−0.539 to 0.101	0.161
Variance OF	−0.264	−0.553 to 0.081	0.131
Skew OF	0.388	−0.058 to 0.642	0.023
Kurtosis OF	0.454	−0.036 to 0.686	0.007

**Fig. 2:**
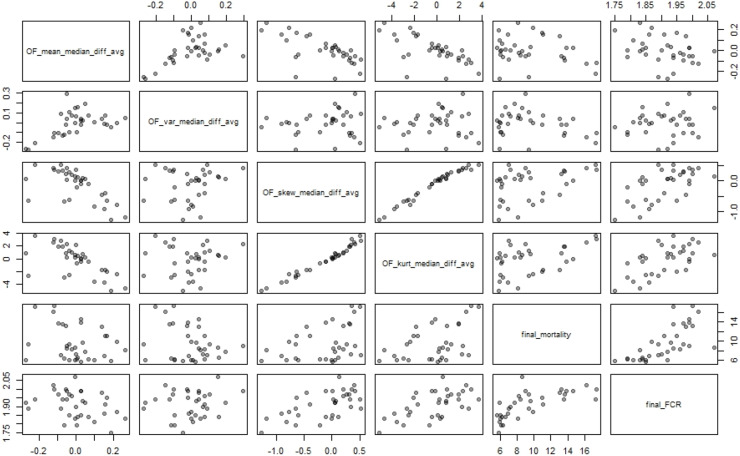
Bivariate scatterplot matrix showing all pairwise relationships among the six variables (the lifetime average scaled deviation of a flock’s activity from the median as measured by each of the optical flow variables, final mortality and FCR). This visualization demonstrates the correlation structure between the variables.

Table 4 and Fig. 2 summarize the correlations between all the variables discussed. Of particular note is the negative correlation between the mean optical flow activity indicator and the skew optical flow activity indicator (Pearson correlation coefficient *r* = −0.636, *P* < 0.001) and that between the mean indicator and the kurtosis indicator (*r* = −0.639, *P* < 0.001), reinforcing the result that more active flocks have lower skew and lower kurtosis**.** There is also a positive Pearson correlation coefficient between FCR and mortality (*r* = 0.698; *P* < 0.001).

**Table 4:** Pearson correlation coefficients between all 6 variables (average lifetime activity indicators based on mean, variance, skew and kurtosis of optical flow, FCR and final mortality).

	Mean OF Activity	Variance OF Activity	Skew OF Activity	Kurtosis OF Activity	Final Mortality	FCR
Mean OF Activity		0.488	−0.636	−0.639	−0.246	−0.326
Variance OF activity			0.137	0.060	−0.264	0.131
Skew OF Activity				0.984	0.388	0.608
Kurtosis OF activity					0.454	0.603
Final Mortality						0.698
FCR

## Discussion

These results directly address farmers’ concerns that improvements to broiler chicken welfare make birds more active and inevitably result in a loss of efficiency. Three different statistical measures of movement all indicate that more active flocks had lower (that is, more efficient) FCR outcomes than less active ones. The most obvious measure – average lifetime level of movement as measured by an activity score based on mean optical flow – shows that on average more active flocks tended to end up with a lower FCR**.** While this negative correlation itself was not statistically significant it should be noted that it was clearly not the positive correlation that would be expected if more active flocks had higher FCR. Our results show clearly that more active flocks are not less efficient as measured by either FCR or % mortality. This is an important finding in its own right and supports results found in pen trials by Shynkaruk et al. (2024) and in farm-scale trials by Kang et al. (2023) who found that enriching the environment of commercial broilers with gradient (variable) lighting resulted both in the birds moving more and in a reduced FCR.

In addition to average (mean) level of movement, we also examined less obvious measures – as indicated by skew and kurtosis of optical flow. These measure different aspects of flock movement and give further clues as to how activity is related to production and welfare outcomes. A positive skew (as observed for all flock-days with non-missing OF skew values), also known as a right-skew, is a type of distribution where the tail is longer on the right with more low values and a few high outliers, meaning that the majority of movement observations cluster at lower values. Such a distribution is typical of commercial broiler flocks where it has been shown by direct observation that individual birds spend up to 90% of their time sitting or lying and only about 10% actively walking (Weeks et al. (2000); Bizeray et al., 2002; Dixon, 2020). At a flock level, this results in a positively skewed movement distribution when measured by optical flow (Roberts, 2012; Dawkins et al., 2017, 2021) – that is, there are many data points showing low levels of movement and only a small number showing very active movements. The finding reported here that a strong predictor of a high FCR was a high positive skew score therefore suggests that FCR increases when larger numbers of birds are inactive.

This conclusion is also consistent with the positive association between kurtosis of optical flow scores and FCR. High kurtosis in a positively skewed distribution (a more peaked distribution with a more pronounced upper tail) has been found empirically to indicate poor welfare and high mortality (Roberts et al., 2012; Dawkins et al., 2017, 2025) and is typical of flocks where active walking is rare and stands out as unusual because of a large relatively immobile majority. A higher kurtosis with higher skew indicates a smaller number of actively walking birds (Dawkins, et al., 2013) and is also associated with higher mortality, higher incidence of hockburn and other negative outcomes (Dawkins et al., 2017, 2021, 2025). The finding reported here that the flocks with lower skew and lower kurtosis activity scores are also the ones with the lowest FCR thus show that good welfare and efficiency can be positively associated.

The health and life expectancy benefits of exercise are now well established in humans through, for example, improved cardiovascular function and musculoskeletal fitness (Garber et al., 2011). In broiler chickens, being active also improves bone strength (Reiter and Bessei, 2009; Pedersen et al., 2020), which could be particularly important for very young chicks at a critical stage in their bone development (Sanchez-Rodriguez et al., 2019). Activity also decreases the incidence and severity of woody breast (Hisasaga and Makagon, 2024) and lack of exercise leads to a higher incidence of leg deformities and walking difficulties (Haye and Simon, 1978; Knowles et al., 2008). Inactive birds also spend more time sitting in contact with litter, which in turn leads to greater incidence of skin lesions including contact dermatitis (Bassler et al., 2013; de Jong et al., 2014).

It is important to point out that our results do not show that that increasing activity will always result in increased efficiency, let alone that it will always improve FCR. What they do show, however, is that under some circumstances increased flock activity can be associated with increased efficiency. The positive correlations between mortality and FCR, particularly using kurtosis as the activity measure, suggests that at least part of this association may be due to the beneficial effects of activity on reduced mortality. The hypothesis that FCR can be improved by focusing on factors that reduce mortality and improve bird health therefore deserves further testing.

It should also be noted that the present study was limited to one farm and one breed, and more studies in commercial settings are needed to understand which welfare improvements have the greatest overall benefits as different conditions (management, environment, genetics) are likely to have different outcomes both on welfare and productivity (Averós and Estevez, 2018). In this context, it is important to stress the need for more on-farm studies. Small-scale pen trials are, of course, an essential first stage in the development of any new welfare improvement. But what works for a few tens or hundreds of chickens in a carefully controlled environment does not necessarily translate into what happens when many thousands of birds are reared on commercial farms. Not only environmental but also financial conditions are completely different, meaning that both welfare and efficiency outcomes may be quite different too.

It follows that if we are to fully understand the relationship between welfare, bird activity and efficiency, we need more farm-scale trials. Improvements to broiler welfare are most likely to be taken up commercially if it can be demonstrated that they are of value not just to the animals and their welfare, but to the farm balance sheet as well.

## Animal use

This research was approved by the University of Oxford Local AWERB (Animal Welfare Ethical Review Board) on 09/08/2019. The Board considered that the work was in accordance with the University’s policy on the use of protected animals for scientific research (Ref. no. APA/1/5/ZOO/NASPA/Dawkins/OpticFlock).

## CRediT authorship contribution statement

**Christl A. Donnelly:** Writing – review & editing, Validation, Methodology, Formal analysis, Conceptualization. **Stephen A. Ellwood:** Writing – review & editing, Validation, Software, Methodology, Investigation, Data curation. **Stephen J. Roberts:** Writing – review & editing, Validation, Software, Conceptualization. **Marian Stamp Dawkins:** Writing – review & editing, Writing – original draft, Validation, Supervision, Project administration, Methodology, Investigation, Funding acquisition, Data curation, Conceptualization.

## Disclosures

The authors declare the following financial interests/personal relationships which may be considered as potential competing interests:

1. Dawkins: financial support was provided by the Foundation for Food & Agriculture Research (FFAR).

2. Dawkins, Ellwood, Roberts, Donnelly: The FFAR SMART Broiler grant that supported this work is co-sponsored by McDonald’s.

3. Dawkins: is a member of McDonald’s Chicken Sustainability Advisory Council and receives travel expenses to meetings but no fee. This appointment has been declared and approved by the Head, Department of Biology, University of Oxford. No pressure has been brought on her or any of the other authors regarding the content or results of this article. The other authors declare that they have no known competing financial interests or personal relationships that could have appeared to influence the work reported in this paper.

## Appendix Supplementary materials

Image, application 1

## Footnotes

Supplementary material associated with this article can be found, in the online version, at doi:10.1016/j.psj.2026.106599.
