# Supplementary material for: Increased activity in broiler chickens is associated with better feed conversion
Source: Poult Sci. 2026 Feb 7;105(5):106599. doi: 10.1016/j.psj.2026.106599 (PMC12925194; doi:10.1016/j.psj.2026.106599)
Supplement: Supplementary file 1 [file mmc1.docx]

SUPPLEMENTARY INFORMATION

**Assessment of data for anomalous values**

We constructed the lifetime average scaled deviation of a flock’s activity from the median based on OF mean, OF variance, OF skew and OF kurtosis. We then considered the minimum and maximum value of each. For OF mean the minimum was -0.27 and the maximum was 0.26. For OF variance the minimum was -0.27 and the maximum was 2.33. For OF skew the minimum was -1.27 and the maximum was 0.52. For OF kurtosis the minimum was -5.14 and the maximum was 3.72. Thus, for all but the OF variance, the minimum and maximum were the same order of magnitude.

Examination of the data revealed that for a single flock, the OF variance for day 33 was 29.4. All other OF values for all flock-days varied between 0.007 and 0.79. We looked at the camera-specific data for this flock on day 33 and found that one camera was showing an OF mean of 0.18 and an OF variance of 0.28, in line with all other flocks. The other camera, however, was showing an OF mean of 0.57 and an OF variance of 58.59. On this basis, we changed a single value in the data, OF variance for day 33 for this flock, to 0.28.

After this change was made, for the lifetime average scaled deviation of a flock’s activity from the median based on OF variance the minimum was -0.27 and the maximum was 0.26.

**Robustness of results**

For the linear regression models with a single predictor (the lifetime average scaled deviation of a flock’s activity from the median based on OF skew or OF kurtosis) and FCR as the outcome variable. We tested the robustness of our results by evaluating the Cook’s distance of each of the 34 data points in the regression. In both regression models there were 3 data points for which the Cook’s distance was greater than three times the average of the Cook’s distances, in other words they were large relative to the rest of the datapoints. They were:

Flock “A” FCR = 1.89 OF skew measure = 0.52 OF kurtosis measure = 2.84

Flock “B” FCR = 2.07 OF skew measure = 0.14 OF kurtosis measure = 0.55

Flock “C” FCR = 1.75 OF skew measure = -1.27 OF kurtosis measure = -5.14

Based on these values, the individual points can be identified in Figure 1 panels a nd b.

We fitted the linear regression models with a single predictor (the lifetime average scaled deviation of a flock’s activity from the median based OF skew or OF kurtosis) and FCR as the outcome variable under 5 conditions:

- With all data points (full dataset),
- Excluding Flock “A”
- Excluding Flock “B”
- Excluding Flock “C”
- Excluding Flocks “A”, “B” and “C”.

Tables A and B give the results in terms of estimates intercept and slope, correlation coefficient and p-value for the null hypothesis of no correlation between the predictor and outcome. The results indicate that the three points identified with relatively high Cook’s distance had little influence on the results obtained.

Table A: Results from the linear regression of with a single predictor (the lifetime average scaled deviation of a flock’s activity from the median based OF skew) and FCR as the outcome variable. (SE = standard error)

| Dataset | Sample size | Correlation | P-value | Intercept | Slope |
| --- | --- | --- | --- | --- | --- |
| Full dataset | 34 | 0.608 (0.340, 0.785) | 0.0001 | 1.92  (SE = 0.011) | 0.093  (SE = 0.021) |
| Excluding Flock A | 33 | 0.638  (0.377, 0.805) | 0.0001 | 1.93  (SE = 0.011) | 0.100  (SE = 0.022) |
| Excluding Flock B | 33 | 0.619  (0.350, 0.794) | 0.0001 | 1.92  (SE = 0.010) | 0.088  (SE = 0.020) |
| Excluding Flock C | 33 | 0.539 (0.240, 0.744) | 0.0012 | 1.92  (SE = 0.011) | 0.084  (SE = 0.023) |
| Excluding Flocks A, B, and C | 31 | 0.581  (0.285, 0.775) | 0.0006 | 1.92  (SE = 0.011) | 0.086  (SE = 0.022) |

Table B: Results from the linear regression of with a single predictor (the lifetime average scaled deviation of a flock’s activity from the median based OF kurtosis) and FCR as the outcome variable. (SE = standard error)

| Dataset | Sample size | Correlation | P-value | Intercept | Slope |
| --- | --- | --- | --- | --- | --- |
| Full dataset | 34 | 0.603 (0.333, 0.782) | 0.0002 | 1.92  (SE= 0.011) | 0.020  (SE = 0.0047) |
| Excluding Flock A | 33 | 0.634  (0.371, 0.802) | 0.0001 | 1.92  (SE = 0.011) | 0.022  (SE = 0.0047) |
| Excluding Flock B | 33 | 0.626 (0.360, 0.798) | 0.0001 | 1.91  (SE = 0.010) | 0.019  (SE = 0.0043) |
| Excluding Flock C | 33 | 0.539 (0.240, 0.744) | 0.0012 | 1.92  (SE = 0.011) | 0.017  (SE = 0.0050) |
| Excluding Flocks A, B, and C | 31 | 0.595  (0.306, 0.784) | 0.0004 | 1.92  (SE = 0.010) | 0.019  (SE = 0.0047) |
